# Broad and Cross-Clade CD4^+^ T-Cell Responses Elicited by a DNA Vaccine Encoding Highly Conserved and Promiscuous HIV-1 M-Group Consensus Peptides

**DOI:** 10.1371/journal.pone.0045267

**Published:** 2012-09-18

**Authors:** Rafael Ribeiro Almeida, Daniela Santoro Rosa, Susan Pereira Ribeiro, Vinicius Canato Santana, Esper Georges Kallás, John Sidney, Alessandro Sette, Jorge Kalil, Edecio Cunha-Neto

**Affiliations:** 1 Laboratory of Clinical Immunology and Allergy-LIM60, Division of Clinical Immunology and Allergy, Department of Medicine, University of São Paulo School of Medicine, São Paulo, Brazil; 2 Heart Institute (InCor), University of São Paulo School of Medicine, São Paulo, Brazil; 3 Institute for Investigation in Immunology-INCT, São Paulo, Brazil; 4 Division of Immunology-Federal University of São Paulo-UNIFESP, São Paulo, Brazil; 5 Center for Infectious Disease, Allergy and Asthma Research, La Jolla Institute for Allergy and Immunology, La Jolla, California, United States of America; National Institute of Allergy and Infectious Diseases, United States of America

## Abstract

T-cell based vaccine approaches have emerged to counteract HIV-1/AIDS. Broad, polyfunctional and cytotoxic CD4^+^ T-cell responses have been associated with control of HIV-1 replication, which supports the inclusion of CD4^+^ T-cell epitopes in vaccines. A successful HIV-1 vaccine should also be designed to overcome viral genetic diversity and be able to confer immunity in a high proportion of immunized individuals from a diverse HLA-bearing population. In this study, we rationally designed a multiepitopic DNA vaccine in order to elicit broad and cross-clade CD4^+^ T-cell responses against highly conserved and promiscuous peptides from the HIV-1 M-group consensus sequence. We identified 27 conserved, multiple HLA-DR-binding peptides in the HIV-1 M-group consensus sequences of Gag, Pol, Nef, Vif, Vpr, Rev and Vpu using the TEPITOPE algorithm. The peptides bound *in vitro* to an average of 12 out of the 17 tested HLA-DR molecules and also to several molecules such as HLA-DP, -DQ and murine IA^b^ and IA^d^. Sixteen out of the 27 peptides were recognized by PBMC from patients infected with different HIV-1 variants and 72% of such patients recognized at least 1 peptide. Immunization with a DNA vaccine (HIVBr27) encoding the identified peptides elicited IFN-γ secretion against 11 out of the 27 peptides in BALB/c mice; CD4^+^ and CD8^+^ T-cell proliferation was observed against 8 and 6 peptides, respectively. HIVBr27 immunization elicited cross-clade T-cell responses against several HIV-1 peptide variants. Polyfunctional CD4^+^ and CD8^+^ T cells, able to simultaneously proliferate and produce IFN-γ and TNF-α, were also observed. This vaccine concept may cope with HIV-1 genetic diversity as well as provide increased population coverage, which are desirable features for an efficacious strategy against HIV-1/AIDS.

## Introduction

The development of an efficacious vaccine against human immunodeficiency virus 1 (HIV-1) still remains as the best long-term approach to control the acquired immunodeficiency syndrome (AIDS) pandemic since resource-poor endemic regions are not able to afford sustained antiretroviral therapy (ART). Clinically tested HIV-1 vaccines have shown no or modest efficacy so far [1], [2]. No vaccine strategy was able to induce broadly neutralizing antibodies and T-cell based vaccines have thus emerged as an alternative to counteract AIDS by limiting both viral transmission and disease progression [3]. Indeed, a recent study using non-human primates (NHP) has demonstrated that vaccine-induced virus-specific effector memory T-cell (TEM) responses can exert a profound early control on highly pathogenic simian immunodeficiency virus (SIV) infection after mucosal challenge, which has given more hope for the development of new T-cell based vaccines against HIV-1 [4].

The breadth of T-cell responses induced against HIV-1 has become a central goal in AIDS vaccine development after the STEP trial failure [1], [5]. In fact, different groups have shown that protection against SIV challenge is strongly associated with induction of either CD4^+^ or CD8^+^ T cells against multiple targets [6]–[9]. Thus, it is important to design novel vaccine platforms in order to broaden T-cell responses against HIV-1.

T-cell based vaccines against HIV-1 are frequently focused on the induction of CD8^+^ T-cell responses, which are known to be responsible for killing virus-infected targets [6], [10]–[12]. However, mounting evidence suggests that CD4^+^ T-cell responses may be important for controlling HIV-1 replication [13]. Although HIV-specific CD4^+^ T cells are preferentially targeted by the virus, the vast majority of these cells remains virus-free at any time *in vivo* [14], which may allow for their antiviral function. In fact, strong virus-specific CD4^+^ T-cell responses have been associated with natural control of HIV-1 infection [15], [16] and cytotoxic CD4^+^ T cells were shown to suppress viral replication in both SIV and HIV-1-infected cells [17], [18]. While the clinical associations of CD4^+^ T-cell responses with HIV-1 control must be carefully interpreted, due to a possible cause-effect issue, the finding that CD4^+^ T-cell depletion reduced vaccine-mediated protection [19] supports a direct role of such cells in HIV-1 immunity. Moreover, some groups have observed the association of vaccine-induced virus-specific CD4^+^ T-cell responses with protection against SIV challenge [7], [20], which further supports a protective role of CD4^+^ T cells. Therefore, it is important to explore the anti-viral immunity exerted by CD4^+^ T cells in order to develop novel vaccines against HIV-1/AIDS. It is possible that the induction of CD4^+^ T cells will be beneficent both due to the help provided to B cells and CD8^+^ T cells as well as due to direct effects on HIV-1-infected targets.

An important concern regarding AIDS vaccine development is how to elicit cellular immune responses to cover multiple HIV-1 circulating variants, which can differ by up to 20% within a subtype and show up to 35% of amino acid divergences between subtypes [21]. Artificially designed M-group consensus sequences display average distances to HIV-1 variants similar to those found intra-subtype and have been considered a potential alternative to circumvent the barrier posed by viral genetic diversity [22]. Indeed, studies have demonstrated that immunogens based on HIV-1 M-group consensus Env were able to provide broad cross-clade T-cell responses in both mice and macaques [23], [24], which suggests an important role for this strategy in HIV-1 vaccines.

The high polymorphism of human leukocyte antigens (HLA), which are responsible for determining the onset of T-cell responses, is also a challenge for vaccine development. It is expected that different HLA-bearing populations respond differently to the same immunogen and this may be decisive for the vaccine success. Vaccines encoding promiscuous peptides, each binding to multiple HLA molecules, may be a solution to this problem by allowing that multiple HLA molecules spread among the population contribute to the induction of broad T-cell responses in most of the immunized individuals. This would confer broader population coverage and enhance vaccine efficacy [25]–[27]. Thus, novel AIDS vaccines should be rationally designed to address both viral and host genetic diversity in order to confer immunity against multiple HIV-1 circulating variants in a population with diverse HLA alleles.

The inclusion of appropriate proteins in HIV-1 vaccines may be crucial for eliciting protective responses. While broad Gag- and Vif-specific responses have been correlated to vaccine-induced protection in SIV-challenged macaques [28], induction of Env-specific CD4^+^ T-cell responses contributed to enhanced SIV replication and accelerated progression to AIDS [29]. Env-specific CD8^+^ T-cell responses were also shown to be a strong predictor for disease progression in HIV-1-infected patients [30]. Furthermore, CD4^+^ T-cell responses targeting Gag and Env-specific epitopes were associated with spontaneous control of viral replication and progression to AIDS, respectively [31].

Recently, our group has designed a DNA vaccine encoding 18 conserved, multiple HLA-DR-binding epitopes from HIV-1 subtype B consensus sequence. This vaccine elicited broad, polyfunctional and long-lasting CD4^+^ T-cell responses in BALB/c and HLA class II transgenic mice [32], [33]. In this work we sought to develop a DNA vaccine that would be able to provide broad CD4^+^ T-cell immunity in a diverse HLA-bearing population, now targeting multiple HIV-1 M-group consensus peptides, potentially cross-reactive to a high proportion of circulating HIV-1 variants. In addition, we excluded Env peptides from our novel vaccine based on the evidence that Env-specific T-cell responses are frequently related to disease progression.

To accomplish our goals, we used the TEPITOPE algorithm [34], which has been successfully applied for *in silico* identification of promiscuous T-cell epitopes in the context of oncology, allergy, autoimmunity and infectious diseases [35]–[40], to scan the HIV-1 M-group consensus sequence. We identified 27 peptides from 7 different HIV-1 proteins (Gag, Pol, Nef, Vif, Vpr, Rev and Vpu), predicted to bind to multiple HLA-DR molecules and conserved among all M-group subtypes. The identified peptides bound *in vitro* to several HLA-DR, -DP and -DQ molecules and also to murine IA^b^ and IA^d^ molecules. The peptides were antigenic in natural infection, being recognized by peripheral blood mononuclear cells (PBMC) from HIV-1-infected patients. Finally, we designed a DNA vaccine (HIVBr27) encoding the 27 peptides *in tandem* and immunized BALB/c mice. HIVBr27 immunization elicited broad, cross-clade and polyfunctional CD4^+^ and CD8^+^ T-cell responses.

## Materials and Methods

### Ethics Statement

The research involving human participants reported in this study was approved by the institutional review board of the University of São Paulo under protocol number 0458/08. Written informed consent was obtained from all subjects.

Mice were housed and manipulated under SPF conditions in the animal care facilities of the Institute of Tropical Medicine, University of São Paulo (IMT/FMUSP). Experiments were performed in accordance to the guidelines of the Ethics committee of University of São Paulo (CAPPesq- HCFMUSP). This study was approved by CAPPesq- HCFMUSP under protocol number 0653/09.

### Identification of HIV-1 M-group Consensus Peptides

We scanned the HIV-1 M-group proteome consensus sequence available at http://www.hiv.lanl.gov/content/sequence/NEWALIGN/align.html with the TEPITOPE algorithm to identify multiple HLA-DR-binding peptides [34]. The TEPITOPE algorithm predicts binding of peptides to 25 distinct HLA-DR molecules based on results from *in vitro* HLA-peptide binding assays. We selected the peptides predicted to bind to at least 18 out of the 25 HLA-DR molecules in the TEPITOPE matrix, using a threshold of 5%. The selected peptides were further analyzed regarding the conservation status when compared to consensus sequences of the HIV-1 subtypes A, B, C, D, F1, F2, G and H. We ended up with 27 peptides (14–24 aa in length) that had each amino acid conserved among at least 50% of the consensus sequences from the HIV-1 subtypes.

### Peptide Synthesis

Peptides were synthesized by solid phase technology using 9-fluorenylmethoxycarbonyl (Fmoc) strategy, with the C terminal carboxyl group in amide form (GL Biochem). Peptide purity and quality were assessed by reverse-phase high performance liquid chromatography and mass spectrometry, and was routinely above 90%.

### HLA Class II and Murine MHC Class II Peptide-binding Assays

Peptide binding assays were performed by incubating purified HLA class II molecules (HLA-DR, -DP and -DQ) or murine IA^b^ and IA^d^ molecules (5–500 nM) with different concentrations of unlabeled peptide inhibitors and 1–10 nM ^125^I-radiolabeled probe peptides for 48 h as previously described [41].

### Construction of a DNA Plasmid Encoding Multiple HIV-1 Peptides

We designed a multiepitopic construct encoding the 27 HIV-1 peptides: protease (53–75), protease (79–95), RT (343–357), RT (354–368), RT (369–391), RT (413–427), RT (431–445), RT (528–546), integrase (28–43), integrase (69–85), integrase (96–113), integrase (216–235), integrase (249–268), p17 (72–90), p17(131–132)/p24(1–18), p24 (33–48), p24 (127–145), p24 (138–153), p24 (182–201), vif (1–15), vif (142–158), rev (9–27), vpr (29–42), vpr (58–80), vpu (13–26), nef (67–87) and nef (133–156). Peptide sequences were codon optimized for mammalian expression and assembled in tandem in the above mentioned order with glycine-proline (GPGPG) spacers at C and N termini to avoid the creation of junctional peptides, which can interfere on processing and presentation [42]. The artificial gene (EZBiolab) was subcloned into pVAX1 vector (Invitrogen) using EcoRI/XhoI sites to generate the HIVBr27 plasmid, which was purified using the Endofree Plasmid Giga Kit (Qiagen) according to manufacturer’s instructions.

### Selection of Variants from HIV-1 M-group Consensus Peptides

The peptides p24(127–145), RT(413–427), RT(528–546) and int(216–235) were aligned with all HIV-1 circulating variants available at http://www.hiv.lanl.gov/content/sequence/QUICK_ALIGN/QuickAlign.html. Sequences from the most frequent variants of each peptide were synthesized to perform immunological analysis.

### Subjects

Cryopreserved peripheral-blood mononuclear cells (PBMC) were obtained from a cohort of HIV-1-infected individuals (n = 25) [43], [44], as well as from healthy volunteers (n = 5), and were used for IFN-γ ELISPOT assay. Infecting viral strains were characterized as previously described [45], [46]. Clinical characteristics of enrolled patients are summarized in the table S1.

### Mice and Immunizations

Six to eight week-old female BALB/c mice were used in this study. Six mice per group were injected intramuscularly, at weeks 0, 2 and 4, with HIVBr27 plasmid or empty pVAX1. Each quadriceps was injected with 50 µL of DNA at a concentration of 1 µg/µL in saline such that each animal received a total of 100 µg of plasmid DNA per immunization. Two weeks after the last DNA immunization, mice were euthanized with CO_2_.

### Spleen Cell Isolation for Immune Assays

Two weeks after the last immunization, mice were euthanized and spleens were removed aseptically. After obtaining single cell suspensions, cells were washed in 10 mL of RPMI 1640. Cells were then resuspended in R-10 (RPMI supplemented with 10% of fetal bovine serum (GIBCO), 2 mM L-glutamine (Life Technologies), 1 mM sodium pyruvate (Life Technologies), 1% vol/vol non-essential amino acids solution (Life Technologies), 1% vol/vol vitamin solution (Life Technologies), 40 mg/mL of Gentamicin, 20 mg/mL of Peflacin and 5×10^−5^ M 2β-mercaptoetanol (Life Technologies). The viability of cells was evaluated using 0.2% Trypan Blue exclusion dye to discriminate between live and dead cells. Cell concentration was estimated with the aid of a Neubauer chamber and adjusted in cell culture medium.

### Detection of IFN-γ Producing Human and Murine Cells by ELISPOT Assay

PBMC (1×10^5^ cells/well) from HIV-1-infected patients and splenocytes (3×10^5^ cells/well) from HIVBr27 or pVAX1 immunized mice were tested for their ability to secrete IFN-γ after *in vitro* stimulation with 5 µM of individual or pooled HIV-1 peptides using ELISPOT assay. The ELISPOT assay was performed using human or murine IFN-γ Becton Dickinson kit according to manufacturer’s instructions. Spots were counted using an AID ELISPOT reader (Autoimmun Diagnostika GmbH). The number of antigen-specific T cells, expressed as spot-forming units (SFU)/10^6^ PMBC or SFU/10^6^ splenocytes, was calculated after subtracting negative control values (medium only). Responses in human ELISPOT assay for each patient were considered positive when ≥50 SFU/10^6^ PBMC [47]. Responses in murine ELISPOT were considered positive when >15 SFU/10^6^ splenocytes, which was calculated as the mean response +3 standard deviations (SD) of splenocytes from pVAX1 immunized mice, stimulated with each peptide.

### CFSE-based Proliferation Assay

To monitor the expansion and proliferation of HIV-1-specific T cells, splenocytes from HIVBr27 or pVAX1 immunized mice were labeled with carboxyfluorescein succinimidyl ester (CFSE) [48]. Briefly, freshly isolated splenocytes were resuspended (50×10^6^/mL) in PBS and labeled with 1.25 µM of CFSE (Molecular Probes) at 37°C for 10 minutes. The reaction was quenched with RPMI 1640 supplemented with 10% FBS and cells were washed before resuspending in RPMI 1640 at a density of 1.5×10^6^/mL. Cells were cultured in 96 well round-bottomed plates (3×10^5^/well in triplicate) for 5 days at 37°C and 5% CO_2_ with medium only or 5 µM of HIV peptides. Positive controls were stimulated with 2.5 µg/mL of Concanavalin A (Sigma). Cells were then harvested, washed with 100 µL of FACS buffer (PBS with 0.5% BSA and 2 mM EDTA) and stained with anti-mouse CD3 phycoerythrin (PE), anti-mouse CD4 peridinin chlorophyll protein (PerCP) and anti-mouse CD8 allophycocyanin (APC) monoclonal antibodies (BD Pharmingen) for 45 minutes at 4°C. Samples were acquired on a FACSCanto flow cytometer (BD Biosciences) and then analyzed using FlowJo software (version 9.0.2, Tree Star). Fifty thousand events were acquired in a live lymphocyte gate. The percent of proliferating CD4^+^ and CD8^+^ CFSE^low^ cells was determined in the CD3^+^ T-cell population. Positive proliferation of T cells was determined as CFSE^low^ T cells > cutoff, which was calculated as median +3 SD of unspecific proliferative responses obtained with splenocytes from pVAX1 immunized mice stimulated with HIV-1 peptides.

**Figure 1:**
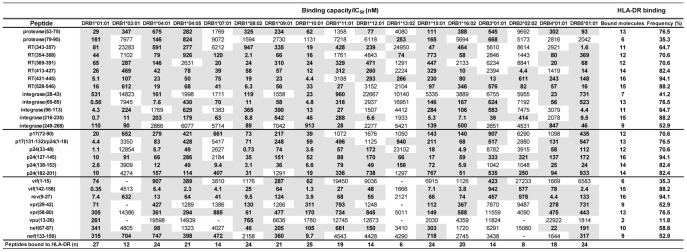
HLA-DR binding assay for M-group consensus peptides. Peptide binding assays were performed by incubating purified HLA-DR molecules (5–500 nM) with different concentrations of unlabeled peptide inhibitors and 1–10 nM ^125^I-radiolabeled probe peptides for 48 h. Significant affinity threshold <1000 nM are shown in gray. A dash represents 50% inhibitory concentration (IC50) >30000 nM.

### Analysis of Polyfunctional HIV-1-specific T-cell Responses

Splenocytes from immunized mice were labeled with CFSE as described above. CFSE-labeled cells were cultured in 96 well round-bottomed plates (5×10^5^/well in triplicate) for 4 days at 37°C and 5% CO_2_ in the presence of medium only or pooled HIV-1 peptides (5 µM). After incubation, cells were restimulated with 2 µg/mL anti-CD28 (BD Pharmingen), 5 µM of pooled HIV-1 peptides and Brefeldin A- GolgiPlugTM (BD Pharmingen) for the last 12 hours. After this period, cells were washed with FACS buffer and surface stained using the monoclonal antibodies anti-CD8-Alexa700 and anti-CD4-PerCP for 30 minutes at 4°C. Cells were then fixed and permeabilized using the Cytofix/CytopermTM kit (BD Pharmingen). Permeabilized cells were washed with Perm/Wash buffer (BD Biosciences) and stained with the monoclonal antibodies anti-CD3-APCCy7, anti-IL-2-PE, anti-TNFα-PECY7 and anti-IFNγ-APC for 30 minutes at 4°C. Following staining, cells were washed twice and resuspended in FACS buffer. All antibodies were from BD Pharmingen. Samples were acquired on a FACSCanto flow cytometer (BD Biosciences) and then analyzed using FlowJo software (version 9.0.2, Tree Star, San Carlo, CA). Cells were gated on forward scatter (FSC)/side scatter (SSC) gate (500,000 events) followed by CD3^+^ gate and subsequent gates on CD4^+^ or CD8^+^ populations. After identification of CD4^+^ and CD8^+^ populations, we designed a gate in each positive population for IFN-γ, TNF-α and IL-2 expression within or not the CFSE^low^ gate. In addition, we used the Boolean gate (FlowJo software version 9.0.2, Tree Star) platform to create several combinations of the three cytokines (IFN-γ, TNF-α and IL-2) within CFSE^low^ population resulting in seven distinct patterns. The percentages of cytokine producing cells within CFSE^low^ population or not were calculated by subtracting background values. For each flow cytometry experiment performed in this paper, unstained and all single-color controls were processed to allow proper compensation.

**Figure 2:**
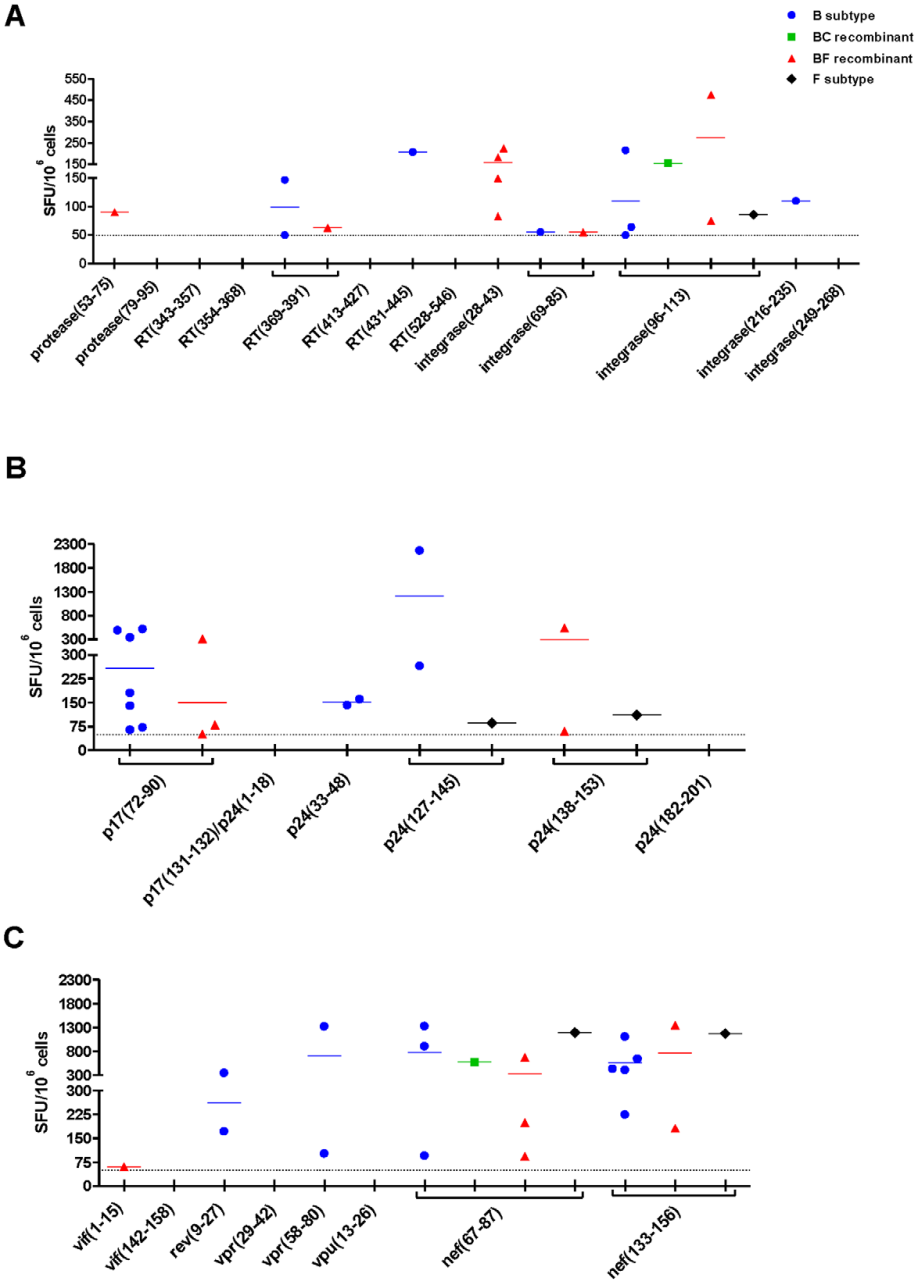
M-group consensus peptides are recognized by PBMC from HIV-1-infected patients. IFN-γ ELISPOT assay was performed to evaluate T-cell responses against the 27 M-group consensus peptides by stimulating PBMC from patients infected with different variants of HIV-1, which are represented by colored symbols. A) IFN-γ secretion against Pol peptides. B) IFN-γ secretion against Gag peptides. C) IFN-γ secretion against Vif, Rev, Vpr, Vpu and Nef peptides. B subtype-infected patients (n = 14), BC recombinant-infected patient (n = 1), BF recombinant-infected patients (n = 9), F subtype-infected patient (n = 1). Dotted lines represent IFN-γ ELISPOT cutoff, which is ≥50 spots/10^6^ cells. Only positive responses are shown in the graphs.

### Statistical Analysis

Statistical significance (p-values) was calculated by using Student’s T test or Two-way ANOVA followed by Bonferroni post test. Statistical analysis and graphical representation of data was performed using GraphPad Prism version 5.0 software.

## Results

### Identification of Conserved, Multiple HLA-DR-binding Peptides in the HIV-1 M-Group Consensus Sequence

The HIV-1 M-group consensus sequence was scanned with the TEPITOPE algorithm in order to identify multiple HLA-DR-binding (promiscuous) peptides. The identified peptides were aligned with consensus sequences from HIV-1 subtypes A1, B, C, D, F1, F2, G and H. Twenty-seven peptides, from 7 different HIV-1 proteins, showing each amino acid conserved among at least 50% of the aligned subtypes consensus sequences were selected (table 1). No Tat peptides were identified with the employed threshold. Env peptides were identified but excluded from further analysis due to the potential prejudice of T-cell responses targeting such protein. We also performed an alignment in order to evaluate whether the 27 peptides from M-group consensus sequence were highly conserved among HIV-1 circulating variants. According to the alignment, most of our peptides had 0–1 amino acid substitutions when compared to all sequences from HIV-1 circulating variants available at the Los Alamos HIV Database (table S2). We performed i*n vitro* HLA-peptide binding assays to confirm the TEPITOPE accuracy and observed that each of the 27 peptides bound to an average of 12 out of the 17 tested HLA-DR molecules and that each HLA-DR molecule bound to an average of 19 out of the 27 peptides (figure 1). The peptides also bound to several HLA-DP and –DQ molecules (table S3 and S4). We also performed murine MHC class II-peptide binding assays and observed that 21 and 6 peptides bound to IA^d^ and IA^b^ molecules, respectively (table 2). Thus, the scanning of HIV-1 M-group consensus sequence with TEPITOPE allowed the identification of highly conserved and promiscuous peptides.

**Figure 3:**
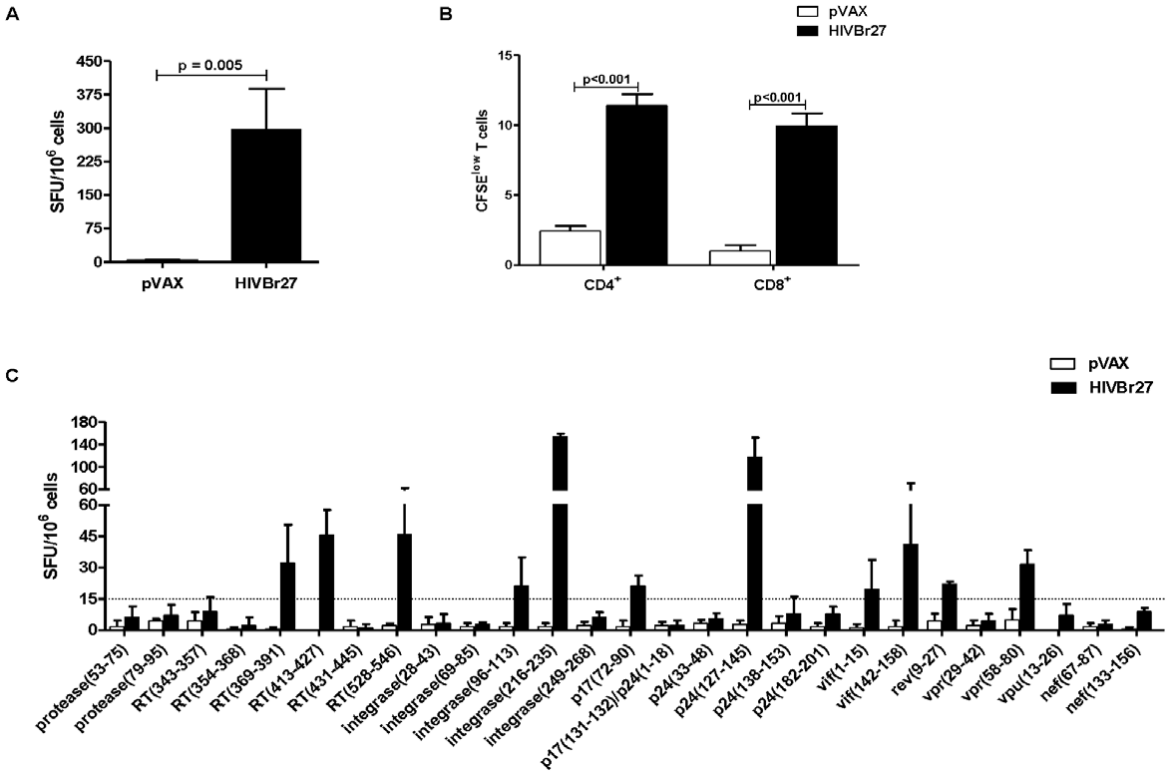
Immunization with HIVBr27 elicits T-cell responses in BALB/c mice. Two weeks after the last immunization with HIVBr27 or empty pVAX1, pooled spleen cells from 6 BALB/c mice were cultured in the presence of individual or pooled HIV-1 peptides (5 µM). Frequency of IFN-γ secreting cells (A) and proliferating CD4^+^ and CD8^+^ T-cells (B) against pooled HIV-1 peptides. C) Frequency of IFN-γ secreting cells against individual HIV-1 peptides. Dotted line represents ELISPOT cutoff values. Data are shown as mean of three independent experiments for ELISPOT assays. Data from proliferation assay are representative of three independent experiments.

**Figure 4:**
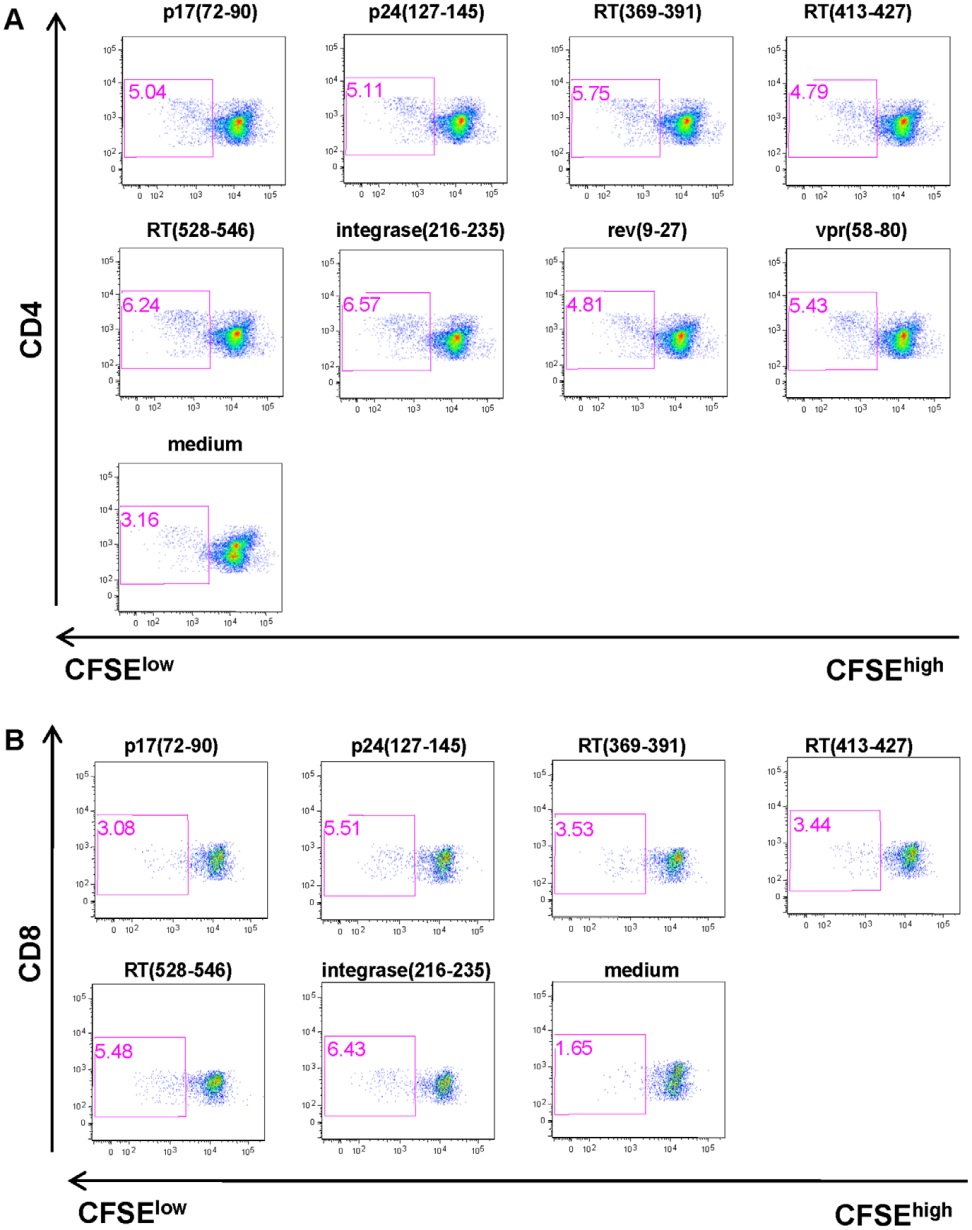
Immunization with HIVBr27 elicits CD4^+^ and CD8^+^ T-cell proliferation against multiple HIV-1 peptides. Two weeks after the last immunization with HIVBr27 or empty pVAX1, pooled spleen cells from 6 BALB/c mice were cultured in the presence of HIV-1 peptides (5 µM) or medium only. Splenocytes were labeled with CFSE (1.25 µM) and cultured for 5 days. After staining with fluorochrome-labeled anti-CD3, -CD4 and -CD8 monoclonal antibodies, cells were analyzed by flow cytometry. CFSE dilution on gated CD3^+^CD4^+^ (A) or CD3^+^CD8^+^ (B) cells was used as readout for antigen-specific proliferation. Data are representative of three independent experiments.

### HIV-1 M-group Consensus Peptides are Antigenic in Natural HIV-1 Infection

To evaluate whether the 27 predicted promiscuous peptides would be antigenic during natural infection we performed an IFN-γ ELISPOT assay using PBMC from twenty-five patients infected with different HIV-1 variants (Table S1). We observed that 7 out of the 13 Pol peptides were recognized at least once (figure 2A). Four out of the 6 Gag peptides were also recognized at least once (figure 2B). Among Vif, Rev, Vpr, Vpu and Nef, we observed recognition of 5 out of the 8 peptides (figure 2C). Overall, we found that 72% of the patients recognized at least 1 peptide and that each patient recognized an average of 2 peptides. In addition, our data shows that 16 out of the 27 peptides (60%) were recognized by HIV-1-infected patients, while no responses were observed in PBMC from healthy individuals (data not shown). Therefore, most of the conserved and promiscuous HIV-1 M-group consensus peptides identified with the TEPITOPE algorithm were showed to be antigenic in natural HIV-1 infection.

**Figure 5:**
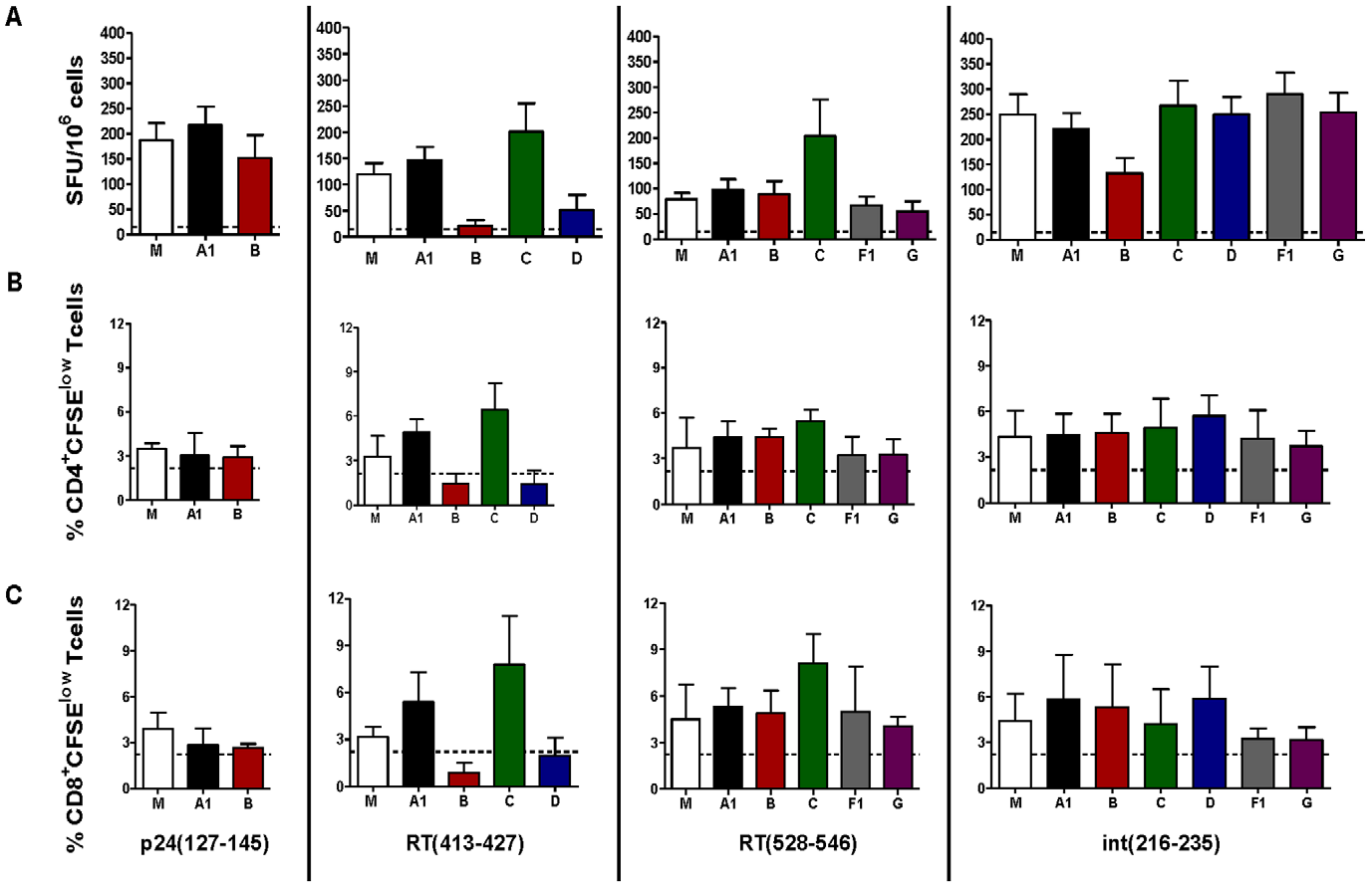
HIVBr27 immunization provides cross-clade immunity. Two weeks after the last immunization with HIVBr27 or empty pVAX1, pooled spleen cells from 6 BALB/c mice were cultured in the presence of HIV-1 M-group consensus peptides (5 µM) (white bars) or their variants (colored bars), from diverse HIV-1 subtypes. Frequency of IFN-γ secreting cells was assessed by ELISPOT assay (A) and proliferative CD4^+^ (B) and CD8^+^ (C) T-cell responses were assessed by CFSE dilution assay. Dotted lines represent ELISPOT or proliferation cutoff, which were calculated as median +3 SD of unspecific responses obtained with splenocytes from pVAX1 immunized mice stimulated with HIV-1 peptides. Data are representative of two independent experiments.

### A DNA Vaccine Encoding 27 Conserved and Promiscuous HIV-1 M-group Consensus Peptides Elicits Broad T-cell Responses in BALB/c Mice

Given the fact that multiple peptides were able to bind to IA^d^ molecules, we chose the BALB/c strain to undergo immunization. To evaluate the immunogenicity of the 27 conserved and promiscuous HIV-1 M-group consensus peptides we designed a DNA vaccine (HIVBr27) encoding the peptides *in tandem*. We assessed the magnitude and breadth of vaccine-elicited HIV-1 specific immune response by IFN-γ ELISPOT and CFSE-based proliferation assays 2 weeks after the last immunization with HIVBr27 or empty pVAX1. HIVBr27 immunization elicited a significantly higher number of IFN-γ secreting cells when compared to empty pVAX1 (figure 3A) and elicited 10% of both CD4^+^ and CD8^+^ proliferating T cells against the pool of 27 HIV-1 peptides (figure 3B). To evaluate the breadth of T-cell responses elicited by HIVBr27, we used individual HIV-1 peptides to stimulate splenocytes *in vitro*. Immunization of BALB/c mice resulted in IFN-γ secretion against 11 peptides (figure 3C) and proliferation of CD4^+^ and CD8^+^ T-cells against 8 and 6 peptides, respectively (figure 4A and 4B). All peptides that stimulated T-cell proliferation also induced IFN-γ secretion. In contrast, pVAX1 immunized mice presented negligible numbers of IFN-γ secreting and proliferating CD4^+^ and CD8^+^ T cells after incubation with the same HIV-1 peptides in all performed experiments. Thus, a DNA vaccine encoding the 27 identified peptides was immunogenic in BALB/c mice, eliciting broad T-cell responses.

**Figure 6:**
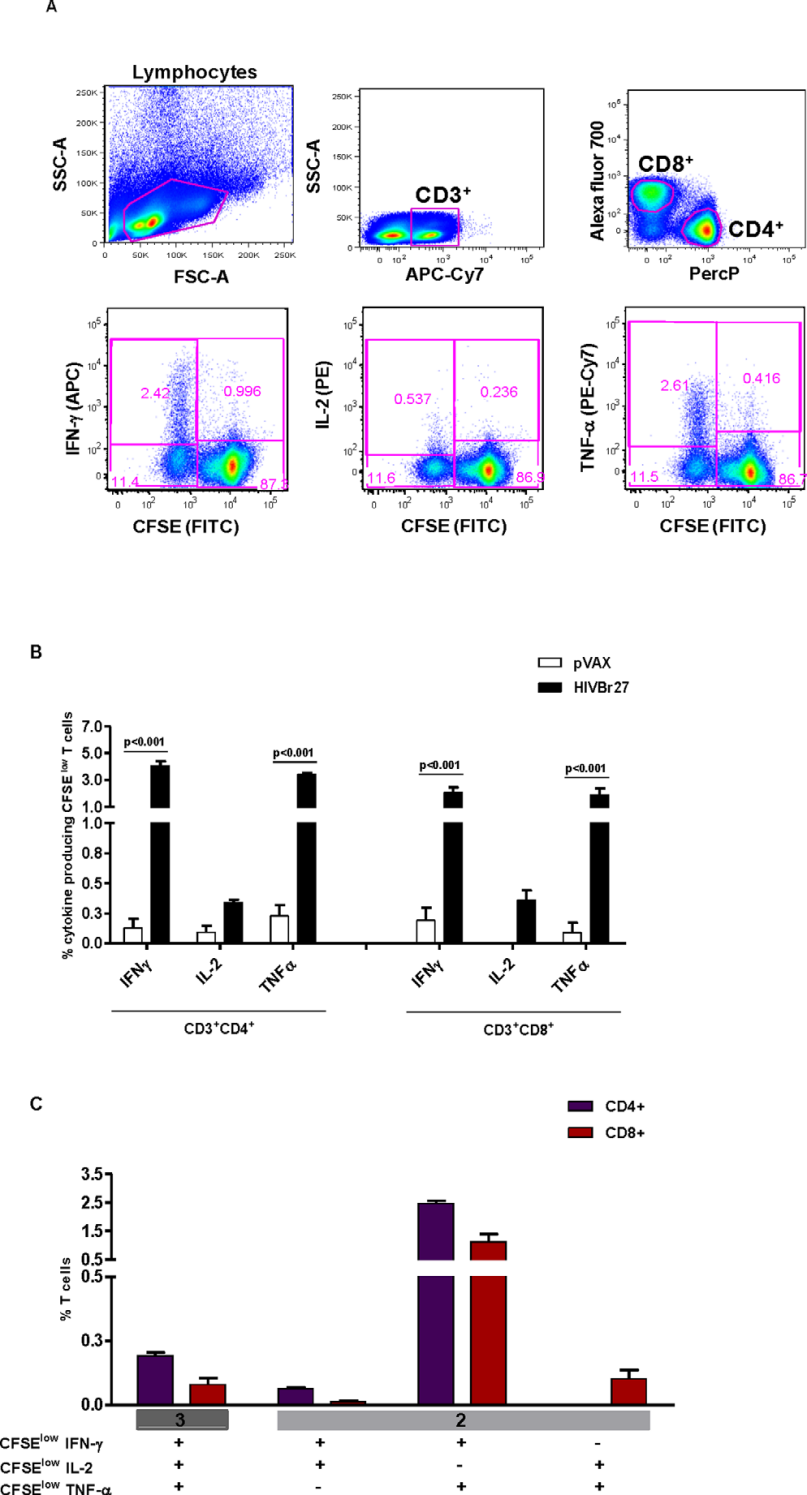
HIVBr27 immunization elicits polyfunctional CD4^+^ and CD8^+^ T cells. Two weeks after the last immunization with HIVBr27 or empty pVAX1, pooled spleen cells from 6 BALB/c mice were collected, labeled with CFSE (1.25 µM) and cultured for 4 days in the presence of pooled HIV-1 peptides (5 µM) or medium only. On day 4, cells were pulsed for 12 hours with pooled peptides in the presence of Brefeldin A and costimulatory antibody (anti-CD28). A) Multiparameter flow cytometry strategy used to determine the frequency of IFN-γ, IL-2 or TNF-α producing CFSE^low^ CD4^+^ and CD8^+^ T cells. B) Frequency of IFN-γ, IL-2 or TNF-α producing CFSE^low^ CD4^+^ (left) and CD8^+^ (right) T-cells. C) Boolean combinations of IFN-γ, IL-2 and TNF-α producing CFSE^low^ CD4^+^ and CD8^+^ T cells from HIVBr27 immunized mice. Background responses detected in negative control tubes (cells stimulated with medium and cells from pVAX1 immunized mice stimulated with pooled peptides) were subtracted from those detected in stimulated samples. Data are representative of three independent experiments.

### HIVBr27 Immunization Provides Cross-clade T-cell Immunity

To evaluate whether HIVBr27 would provide cross-clade immunity, we synthesized the most frequent variant sequences of the 4 most immunogenic HIVBr27 encoded peptides, which are RT(413–427), RT(528–546), int(216–235) and p24(127–145) (table S5), and used them to stimulate splenocytes from immunized BALB/c mice. HIVBr27 immunization elicited comparable frequencies of IFN-γ secreting cells against M-group peptides RT(528–546), int(216–235) and p24(127–145) when compared to most of their variants, while only IFN-γ secretion against RT(413–427) B and D variants was reduced (figure 5A). The same pattern was observed for CD4^+^ and CD8^+^ T-cell proliferation (figure 5B and 5C). We evaluated the avidity of T-cell responses against the 4 HIVBr27 encoded peptides and their variants in IFN-γ ELISPOT assay by serial peptide dilution and observed similar responses among all peptides in a concentration range from 5 µM to 5 pM (data not shown). Besides, most of the variant sequences kept the promiscuous profile observed in their parent peptides, according to the TEPITOPE algorithm (table S5). Taken together, these results suggest that HIVBr27 immunization elicits cross-clade T-cell responses of similar avidity, towards the most frequent variants of the M-group consensus peptides.

### HIVBr27 Immunization Elicits Polyfunctional CD4^+^ and CD8^+^ T-cell Responses

To assess the functional profile of both CD4^+^ and CD8^+^ T-cell vaccine-induced responses we used multiparameter flow cytometry. The gating strategy is outlined in figure 6A. HIVBr27 immunization elicited higher frequencies of IFN-γ and TNF-α producing CFSE^low^ CD4^+^ T cells (4% and 3.4%, respectively) than CD8^+^ T cells (2% and 1.8%, respectively) against pooled HIV-1 peptides (figure 6B). It is of note that there were lower frequencies of IL-2 producing CFSE^low^ CD4^+^ and CD8^+^ T-cells (0.34% and 0.36%, respectively) compared to other cytokine producing cells (figure 6B). To analyze whether HIVBr27 elicits polyfunctional T cells, Boolean combinations of cytokine-producing CFSE^low^ CD4^+^ and CD8^+^ T cells were generated. HIVBr27 immunization elicited about 0.2% of IFN-γ^+^IL-2^+^TNF-α^+^ CFSE^low^ CD4^+^ T cells and less than 0.1% of IFN-γ^+^IL-2^+^TNF-α^+^ CFSE^low^ CD8^+^ T cells (figure 6C). We observed higher frequencies of both CFSE^low^ CD4^+^ and CD8^+^ T cells able to produce the combination of IFN-γ and TNF-α (2.5% and 1.12%, respectively) compared to triple cytokine producing CFSE^low^ T cells (figure 6C). Overall, these data showed that HIVBr27 immunization can elicit polyfunctional CD4^+^ and CD8^+^ T cells that simultaneously proliferate and produce effector cytokines.

## Discussion

In the present study, we designed a DNA vaccine encoding 27 highly conserved and promiscuous peptides from the HIV-1 M-group consensus sequence. This vaccine (HIVBr27) elicited broad, cross-clade and polyfunctional CD4^+^ and CD8^+^ T-cell responses in BALB/c mice. The 27 encoded peptides bound *in vitro* to multiple HLA-DR, -DP, -DQ and to murine IA^b^ and IA^d^ molecules. Furthermore, the peptides were shown to be antigenic in natural infection, being recognized by PBMC from patients infected with different HIV-1 variants.

The 27 peptides identified with the TEPITOPE algorithm [34] bound *in vitro* to an average of 12 out of the 17 tested HLA-DR molecules and to several HLA-DP and -DQ molecules, showing that our peptides are highly promiscuous and that TEPITOPE is accurate in predicting promiscuous HLA-peptide binding. Moreover, each tested HLA-DR molecule bound on average to 19 out of the 27 peptides, which indicates that a vaccine encoding such peptides would have potential to induce T-cell responses against multiple targets in a wide proportion of the population. Notably, all peptides bound to at least one HLA class II molecule associated with AIDS protection, such as HLA-DRB1*0101, -DRB3*0202, -DRB1*13 and -DQB1*06 [49], [50], which suggests that those peptides may be involved in protective immunity against HIV-1. The 27 peptides were also shown to be highly conserved when compared to sequences from all HIV-1 circulating variants available at the Los Alamos HIV Database, indicating that the identified peptides, or sequences highly homologous to them, are broadly represented across several HIV-1 subtypes.

The peptides identified in this study showed to be antigenic during natural infection since we observed that 72% of the HIV-1-infected patients recognized at least one peptide and that 16 out of the 27 peptides were recognized. The responses were similar to those previously found by our group among clinically matched HIV-1-infected patients, towards HIV-1 B subtype consensus peptides [51]. It is of note that T-cell responses against M-group consensus Gag peptides were previously observed in HIV-1-infected patients [52]. In this study, 34% of the 15-mer M-group consensus peptides were recognized by HIV-1 B subtype-infected patients, while we observed recognition of 44% of our peptides by patients infected with the same subtype. Considering only our Gag peptides, we observed 50% of recognition by HIV-1 B subtype-infected patients, suggesting that our TEPITOPE-selected conserved and promiscuous HIV-1 M-group consensus peptides were more frequently recognized than the 15-mer peptides employed in the previous study.

HIVBr27 immunization led to broad T-cell responses in BALB/c mice, with recognition of 11 peptides in IFN-γ ELISPOT assay, which is a desirable result since broad T-cell responses have been associated with protective vaccines [6], [7], [20]. We extended our immunological analysis and also observed broad CD4^+^ and CD8^+^ T-cell proliferation, against 8 and 6 peptides, respectively. Elicitation of HIV-1-specific proliferating CD8^+^ T cells with this vaccine designed to elicit CD4^+^ T-cell responses may have occurred due to the fact that long peptides can harbor CD8^+^ T-cell targets [53]. In addition, 24 out of the 27 peptides were predicted to bind to MHC class I molecules from BALB/c mice, according to the Pred^Balb/c^ algorithm [54], which could explain the potential of HIVBr27 to induce CD8^+^ T-cell responses. The broad T-cell response observed in BALB/c mice, despite the fact that the peptides were selected for promiscuous HLA-DR binding, is supported by results from the *in vitro* MHC-peptide binding assay, in which 21 out of the 27 peptides effectively bound to IA^d^ molecules. Moreover, previous studies have already reported cross-species recognition of peptides identified with TEPITOPE, probably by the fact that such algorithm may select for promiscuous peptides that share MHC class II binding motifs similar to many other human and non-human MHC class II molecules [33], [37], [55].

HIVBr27 immunization elicited comparable T-cell responses against M-group consensus peptides and their respective high-frequency variants, providing evidence that HIVBr27 is able to elicit cross-clade immunity across several HIV-1 subtypes, which is thought to be essential to afford protection against the global diversity of HIV-1. Moreover, we believe that our approach has brought significant improvement on the HIV-1 M-group consensus-based vaccine strategy, since we designed a vaccine encoding highly conserved and promiscuous CD4^+^ T-cell epitopes from 7 different viral genes, while no other study had addressed all these features together. Previous HIV-1 M-group consensus-based vaccines only focused on inducing cross-clade immune responses against Env [23], [24], [56]. These studies were able to elicit broader cross-subtype neutralizing antibodies and broader cross-clade T-cell responses when compared to immunogens based on single HIV-1 subtypes. However, their immunogens failed to include other HIV-1 proteins, as was done in HIVBr27. We conceive that multiepitopic approaches as HIVBr27 may facilitate the emergence of each peptide as individually immunogenic [25], [42], [57], being more attractive to broaden T-cell responses against HIV-1. Indeed, it has been shown that immunization with multiple peptides increased the breadth of the T-cell responses, as compared to those induced by whole proteins [58], [59].

Given the frequent association of either Env-specific CD4^+^ or CD8^+^ T-cell responses with progression to AIDS [29]–[31], [60], [61], it is possible to speculate that an Env-based T-cell immunogen may not provide protection against AIDS progression. However, results from the RV144 trial showing predominant anti-Env CD4 responses [2], and the correlation between Env-specific antibodies and protection against HIV-1 infection [62] suggest that a vaccine able to elicit strong CD4^+^ T-cell responses against Env would be valuable in order to improve antibody-mediated protection. The higher risk of HIV-1 infection observed in vaccinated subjects from the Step study may raise questions about the association of T-cell responses specific to other viral proteins with susceptibility of infection. However, even after extended analysis, it still seems to be an event related to the prior adenovirus 5-specific immunity [63]. Thus, it is important to carefully evaluate the inclusion of Env in HIV-1 vaccine formulations.

Different approaches have been suggested to overcome HIV-1 genetic diversity. Ad26-vectored mosaic vaccines comprising Gag, Pol and Env were found to be more immunogenic than a similar vaccine based on M-group consensus sequence in rhesus macaques [64]. However, it is important to note that the M-group vaccine studied by the authors was based on whole HIV-1 proteins, unlike our approach. Another strategy proposed to elicit cross-clade T-cell responses was based on a chimaeric protein composed of conserved regions of Pol, Gag, Vif and Env from HIV-1 A, B, C and D subtypes [65]. This approach induced significant CD8^+^ T-cell responses in immunized mice, but limited CD4^+^ T-cell responses compared to those induced with HIVBr27 immunization. Indeed, HIVBr27 is the first vaccine specifically aimed to elicit broad and cross-clade CD4^+^ T-cell responses against HIV-1. Thus, we hypothesize that HIVBr27 may confer direct CD4^+^ T-cell-mediated antiviral immunity, as well as provide help for CD8^+^ T-cell-based vaccines, since CD4^+^ T cells are required for both enhancement of virus-specific CD8^+^ T-cell effector function and mobilization of these cells to infected tissues [66]–[68].

HIVBr27 immunization was also able to elicit high frequencies of both polyfunctional CD4^+^ and CD8^+^ T cells, which simultaneously proliferate and produce effector cytokines such as IFN-γ and TNF-α. We observed a low but detectable frequency of CD4^+^ T cells able to proliferate and produce IFN-γ, IL-2 and TNF-α. These results indicate that HIVBr27 has potential to induce T-cell responses with a functional profile related to natural control of viral replication and non progression to AIDS, as previously observed in HIV-1-infected patients [50], [69]–[71] and vaccine-mediated protection in NHP models [9], [72].

We hereby demonstrate that immunization with the HIVBr27 vaccine encoding multiple conserved and promiscuous HIV-1 M-group consensus peptides is able to elicit broad, cross-clade and polyfunctional T-cell responses. This vaccine concept may cope with HIV-1 genetic diversity as well as provide increased population coverage, which are desirable features for an efficacious strategy against HIV-1/AIDS.

## Supporting Information

Table S1 **Clinical characteristics of HIV-1-infected patients.**
(XLS)

Table S2 **Frequency of HIV-1 circulating variants according to the number of amino acid substitutions compared to M-group consensus peptides.** M-group consensus peptides were aligned with HIV-1 circulating variants using all available sequences at http://www.hiv.lanl.gov/content/sequence/QUICK_ALIGN/QuickAlign.html. For each peptide, the highest frequency of variants is shown in bold.
(XLS)

Table S3 **HLA-DP binding assay for M-group consensus peptides.** Significant affinity threshold <1000 nM are shown in bold. A dash represents 50% inhibitory concentration (IC50) >30000 nM.
(XLS)

Table S4 **HLA-DQ binding assay for M-group consensus peptides.** Significant affinity threshold <1000 nM are shown in bold. A dash represents 50% inhibitory concentration (IC50) >30000 nM.
(XLS)

Table S5 **HIV-1 M-group consensus peptides and variant sequences.** The most frequent variants of the 4 most immunogeic M-group consensus peptides were obtained at http://www.hiv.lanl.gov/content/sequence/QUICK_ALIGN/QuickAlign.html. The percentage of HLA-DR molecules predicted to bind to each sequence according to TEPITOPE (threshold of 5%) is also shown.
(XLS)

## References

[1] Buchbinder SP, Mehrotra DV, Duerr A, Fitzgerald DW, Mogg R, et al (2008) Efficacy assessment of a cell-mediated immunity HIV-1 vaccine (the Step Study): a double-blind, randomised, placebo-controlled, test-of-concept trial. Lancet 372: 1881–1893. 19012954 10.1016/S0140-6736(08)61591-3 PMC2721012

[2] Rerks-Ngarm S, Pitisuttithum P, Nitayaphan S, Kaewkungwal J, Chiu J, et al (2009) Vaccination with ALVAC and AIDSVAX to Prevent HIV-1 Infection in Thailand. New England Journal of Medicine 361: 2209–2220. 19843557 10.1056/NEJMoa0908492

[3] McElrath MJ, Haynes BF (2010) Induction of Immunity to Human Immunodeficiency Virus Type-1 by Vaccination. Immunity 33: 542–554. 21029964 10.1016/j.immuni.2010.09.011 PMC3031162

[4] Hansen SG, Ford JC, Lewis MS, Ventura AB, Hughes CM, et al (2011) Profound early control of highly pathogenic SIV by an effector memory T-cell vaccine. Nature 473: 523–U270. 21562493 10.1038/nature10003 PMC3102768

[5] Corey L, McElrath MJ, Kublin JG (2009) Post-Step modifications for research on HIV vaccines. Aids 23: 3–8. 19050380 10.1097/QAD.0b013e32830e6d6d PMC2720076

[6] Wilson NA, Keele BF, Reed JS, Piaskowski SM, MacNair CE, et al (2009) Vaccine-Induced Cellular Responses Control Simian Immunodeficiency Virus Replication after Heterologous Challenge. Journal of Virology 83: 6508–6521. 19403685 10.1128/JVI.00272-09 PMC2698536

[7] Hansen SG, Vieville C, Whizin N, Coyne-Johnson L, Siess DC, et al (2009) Effector memory T cell responses are associated with protection of rhesus monkeys from mucosal simian immunodeficiency virus challenge. Nature Medicine 15: 293–299. 10.1038/nm.1935 PMC2720091 19219024

[8] Hel Z, Tsai WP, Tryniszewska E, Nacsa J, Markham PD, et al (2006) Improved vaccine protection from simian AIDS by the addition of nonstructural simian immunodeficiency virus genes. Journal of Immunology 176: 85–96. 10.4049/jimmunol.176.1.85 16365399

[9] Liu JY, O’Brien KL, Lynch DM, Simmons NL, La Porte A, et al (2009) Immune control of an SIV challenge by a T-cell-based vaccine in rhesus monkeys. Nature 457: 87–91. 18997770 10.1038/nature07469 PMC2614452

[10] Wilson CC, McKinney D, Anders M, MaWhinney S, Forster J, et al (2003) Development of a DNA vaccine designed to induce cytotoxic T lymphocyte responses to multiple conserved epitopes in HIV-1. Journal of Immunology 171: 5611–5623. 10.4049/jimmunol.171.10.5611 14607970

[11] Barouch DH, Santra S, Schmitz JE, Kuroda MJ, Fu TM, et al (2000) Control of viremia and prevention of clinical AIDS in rhesus monkeys by cytokine-augmented DNA vaccination. Science 290: 486–492. 11039923 10.1126/science.290.5491.486

[12] Egan MA, Charini WA, Kuroda MJ, Schmitz JE, Racz P, et al (2000) Simian immunodeficiency virus (SIV) gag DNA-vaccinated rhesus monkeys develop secondary cytotoxic T-lymphocyte responses and control viral replication after pathogenic SIV infection. Journal of Virology 74: 7485–7495. 10906202 10.1128/jvi.74.16.7485-7495.2000 PMC112269

[13] Porichis F, Kaufmann DE (2011) HIV-specific CD4 T cells and immune control of viral replication. Current Opinion in Hiv and Aids 6: 174–180. 21502921 10.1097/COH.0b013e3283454058 PMC3265969

[14] Douek DC, Brenchley JM, Betts MR, Ambrozak DR, Hill BJ, et al (2002) HIV preferentially infects HIV-specific CD4(+) T cells. Nature 417: 95–98. 11986671 10.1038/417095a

[15] Gloster SE, Newton P, Cornforth D, Lifson JD, Williams I, et al (2004) Association of strong virus-specific CD4 T cell responses with efficient natural control of primary HIV-1 infection. Aids 18: 749–755. 15075509 10.1097/00002030-200403260-00005

[16] Rosenberg ES, Billingsley JM, Caliendo AM, Boswell SL, Sax PE, et al (1997) Vigorous HIV-1-specific CD4(+) T cell responses associated with control of viremia. Science 278: 1447–1450. 9367954 10.1126/science.278.5342.1447

[17] Zheng N, Fujiwara M, Ueno T, Oka S, Takiguchi M (2009) Strong Ability of Nef-Specific CD4(+) Cytotoxic T Cells To Suppress Human Immunodeficiency Virus Type 1 (HIV-1) Replication in HIV-1-Infected CD4(+) T Cells and Macrophages. Journal of Virology 83: 7668–7677. 19457989 10.1128/JVI.00513-09 PMC2708625

[18] Sacha JB, Giraldo-Vela JP, Buechler MB, Martins MA, Maness NJ, et al (2009) Gag- and Nef-specific CD4(+) T cells recognize and inhibit SIV replication in infected macrophages early after infection. Proceedings of the National Academy of Sciences of the United States of America 106: 9791–9796. 19478057 10.1073/pnas.0813106106 PMC2687996

[19] Vaccari M, Mattapallil J, Song K, Tsai WP, Hryniewicz A, et al (2008) Reduced protection from simian immunodeficiency virus SIVmac251 infection afforded by memory CD8(+) T cells induced by vaccination during CD4(+) T-cell deficiency. Journal of Virology 82: 9629–9638. 18667509 10.1128/JVI.00893-08 PMC2546957

[20] Gauduin MC, Yu Y, Barabasz A, Carville A, Piatak M, et al (2006) Induction of a virus-specific effector-memory CD4(+) T cell response by attenuated SIV infection. Journal of Experimental Medicine 203: 2661–2672. 17116733 10.1084/jem.20060134 PMC2118155

[21] Gaschen B, Taylor J, Yusim K, Foley B, Gao F, et al (2002) AIDS - Diversity considerations in HIV-1 vaccine selection. Science 296: 2354–2360. 12089434 10.1126/science.1070441

[22] Rolland M, Nickle DC, Mullins JI (2007) HIV-1 group M conserved elements vaccine. Plos Pathogens 3: 1551–1555. 10.1371/journal.ppat.0030157 PMC2098811 18052528

[23] Weaver EA, Lu ZJ, Camacho ZT, Moukdar F, Liao HX, et al (2006) Cross-subtype T-cell immune responses induced by a human immunodeficiency virus type 1 group M consensus env immunogen. Journal of Virology 80: 6745–6756. 16809280 10.1128/JVI.02484-05 PMC1489064

[24] Santra S, Korber BT, Muldoon M, Barouch DH, Nabel GJ, et al (2008) A centralized gene-based HIV-1 vaccine elicits broad cross-clade cellular immune responses in rhesus monkeys. Proceedings of the National Academy of Sciences of the United States of America 105: 10489–10494. 18650391 10.1073/pnas.0803352105 PMC2483234

[25] Depla E, Van der Aa A, Livingston BD, Crimi C, Allosery K, et al (2008) Rational design of a multiepitope vaccine encoding T-Lymphocyte epitopes for treatment of chronic hepatitis B virus infections. Journal of Virology 82: 435–450. 17942551 10.1128/JVI.01505-07 PMC2224390

[26] Kovjazin R, Volovitz I, Kundel Y, Rosenbaum E, Medalia G, et al (2011) ImMucin: A novel therapeutic vaccine with promiscuous MHC binding for the treatment of MUC1-expressing tumors. Vaccine 29: 4676–4686. 21570434 10.1016/j.vaccine.2011.04.103

[27] Depil S, Morales O, Castelli FA, Delhem N, Francois V, et al (2007) Determination of a HLA II promiscuous peptide cocktail as potential vaccine against EBV latency II malignancies. Journal of Immunotherapy 30: 215–226. 17471168 10.1097/01.cji.0000211338.99137.4f

[28] Martins MA, Wilson NA, Reed JS, Ahn CD, Klimentidis YC, et al (2010) T-Cell Correlates of Vaccine Efficacy after a Heterologous Simian Immunodeficiency Virus Challenge. Journal of Virology 84: 4352–4365. 20164222 10.1128/JVI.02365-09 PMC2863752

[29] Staprans SI, Barry AP, Silvestri G, Safrit JT, Kozyr N, et al (2004) Enhanced SIV replication and accelerated progression to AIDS in macaques primed to mount a CD4 T cell response to the SIV envelope protein. Proceedings of the National Academy of Sciences of the United States of America 101: 13026–13031. 15326293 10.1073/pnas.0404739101 PMC516468

[30] Pettersen FO, Tasken K, Kvale D (2010) Combined Env- and Gag-specific T cell responses in relation to programmed death-1 receptor and CD4+T cell loss rates in human immunodeficiency virus-1 infection. Clinical and Experimental Immunology 161: 315–323. 20491784 10.1111/j.1365-2249.2010.04179.x PMC2909414

[31] Ranasinghe S, Flanders M, Cutler S, Soghoian DZ, Ghebremichael M, et al (2012) HIV-Specific CD4 T Cell Responses to Different Viral Proteins Have Discordant Associations with Viral Load and Clinical Outcome. Journal of Virology 86: 277–283. 22031937 10.1128/JVI.05577-11 PMC3255877

[32] Ribeiro SP, Rosa DS, Fonseca SG, Mairena EC, Postol E, et al.. (2010) A Vaccine Encoding Conserved Promiscuous HIV CD4 Epitopes Induces Broad T Cell Responses in Mice Transgenic to Multiple Common HLA Class II Molecules. Plos One 5. 10.1371/journal.pone.0011072 PMC2884037 20552033

[33] Rosa DS, Ribeiro SP, Almeida RR, Mairena EC, Postol E, et al.. (2011) A DNA Vaccine Encoding Multiple HIV CD4 Epitopes Elicits Vigorous Polyfunctional, Long-Lived CD4(+) and CD8(+) T Cell Responses. Plos One 6. 10.1371/journal.pone.0016921 PMC3037933 21347287

[34] Sturniolo T, Bono E, Ding JY, Raddrizzani L, Tuereci O, et al (1999) Generation of tissue-specific and promiscuous HLA ligand databases using DNA microarrays and virtual HLA class II matrices. Nature Biotechnology 17: 555–561. 10.1038/9858 10385319

[35] Schroers R, Huang XF, Hammer J, Zhang JW, Chen SY (2002) Identification of HLA DR7-restricted epitopes from human telomerase reverse transcriptase recognized by CD4+T-helper cells. Cancer Research 62: 2600–2605. 11980655

[36] de Lalla C, Sturniolo T, Abbruzzese L, Hammer J, Sidoli A, et al (1999) Cutting edge: Identification of novel T cell epitopes in Lol p5a by computational prediction. Journal of Immunology 163: 1725–1729. 10438899

[37] Rosa DS, Iwai LK, Tzelepis F, Bargieri DY, Medeiros MA, et al (2006) Immunogenicity of a recombinant protein containing the Plasmodium vivax vaccine candidate MSP1(19) and two human CD4(+) T-cell epitopes administered to non-human primates (Callithrix jacchus jacchus). Microbes and Infection 8: 2130–2137. 16797207 10.1016/j.micinf.2006.03.012

[38] Iwai LK, Yoshida M, Sadahiro A, da Silva WR, Marin ML, et al (2007) T-cell recognition of Paracoccidioides brasiliensis gp43-derived peptides in patients with paracoccidioidomycosis and healthy individuals. Clinical and Vaccine Immunology 14: 474–476. 17329443 10.1128/CVI.00458-06 PMC1865602

[39] Bian HJ, Hammer J (2004) Discovery of promiscuous HLA-II-restricted T cell epitopes with TEPITOPE. Methods 34: 468–475. 15542373 10.1016/j.ymeth.2004.06.002

[40] Iwai LK, Yoshida M, Sidney J, Shikanai-Yasuda MA, Goldberg AC, et al (2003) In silico prediction of peptides binding to multiple HLA-DR molecules accurately identifies immunodominant epitopes from gp43 of Paracoccidioides brasiliensis frequently recognized in primary peripheral blood mononuclear cell responses from sensitized individuals. Molecular Medicine 9: 209–219. 15208742 PMC1430984

[41] Sidney J, Southwood S, Oseroff C, del Guercio MF, Sette A, et al.. (2001) Measurement of MHC/peptide interactions by gel filtration. Current protocols in immunology Chapter 18:Unit 18.3. 10.1002/0471142735.im1803s31 18432745

[42] Livingston B, Crimi C, Newman M, Higashimoto Y, Appella E, et al (2002) A rational strategy to design multiepitope immunogens based on multiple th lymphocyte epitopes. Journal of Immunology 168: 5499–5506. 10.4049/jimmunol.168.11.5499 12023344

[43] Batista MD, Ferreira S, Sauer MM, Tomiyama H, Maidana Giret MT, et al.. (2009) High Human Herpesvirus 8 (HHV-8) Prevalence, Clinical Correlates and High Incidence among Recently HIV-1-Infected Subjects in Sao Paulo, Brazil. Plos One 4. 10.1371/journal.pone.0005613 PMC2682704 19479040

[44] Kallas EG, Bassichetto KC (2004) Oliveira (2004) Establishment of the serologic testing algorithm for recent human immunodeficiency virus (HIV) seroconversion (STARHS) strategy in the city of São Paulo, Brazil. The Brazilian Journal of Infectious Diseases 8: 8. 10.1590/s1413-86702004000600003 15880230

[45] Sa-Filho D, Kallas EG, Sanabani S, Sabino E, Sucupira MC, et al (2007) Characterization of the full-length human immunodeficiency virus-1 genome from recently infected subjects in Brazil. Aids Research and Human Retroviruses 23: 1087–1094. 17919103 10.1089/aid.2006.0173

[46] Sanabani SS, de Souza Pastena ER, da Costa AC, Martinez VP, Kleine-Neto W, et al.. (2011) Characterization of Partial and Near Full-Length Genomes of HIV-1 Strains Sampled from Recently Infected Individuals in Sao Paulo, Brazil. Plos One 6. 10.1371/journal.pone.0025869 PMC3193532 22022460

[47] Samri A, Durier C, Urrutia A, Sanchez I, Gahery-Segard H, et al (2006) Evaluation of the interlaboratory concordance in quantification of human immunodeficiency virus-specific T cells with a gamma interferon enzyme-linked immunospot assay. Clinical and Vaccine Immunology 13: 684–697. 16760328 10.1128/CDLI.00387-05 PMC1489560

[48] Quah BJC, Warren HS, Parish CR (2007) Monitoring lymphocyte proliferation in vitro and in vivo with the intracellular fluorescent dye carboxyfluorescein diacetate succinimidyl ester. Nature Protocols 2: 2049–2056. 17853860 10.1038/nprot.2007.296

[49] Lacap PA, Huntington JD, Luo M, Nagelkerke NJD, Bielawny T, et al (2008) Associations of human leukocyte antigen DRB with resistance or susceptibility to HIV-1 infection in the Pumwani Sex Worker Cohort. Aids 22: 1029–1038. 18520346 10.1097/QAD.0b013e3282ffb3db

[50] Ferre AL, Hunt PW, McConnell DH, Morris MM, Garcia JC, et al (2010) HIV Controllers with HLA-DRB1*13 and HLA-DQB1*06 Alleles Have Strong, Polyfunctional Mucosal CD4(+) T-Cell Responses. Journal of Virology 84: 11020–11029. 20719952 10.1128/JVI.00980-10 PMC2953185

[51] Fonseca SG, Coutinho-Silva A, Fonseca LAM, Segurado AC, Moraes SL, et al (2006) Identification of novel consensus CD4 T-cell epitopes from clade B HIV-1 whole genome that are frequently recognized by HIV-1 infected patients. Aids 20: 2263–2273. 17117012 10.1097/01.aids.0000253353.48331.5f

[52] Bansal A, Gough E, Ritter D, Wilson C, Mulenga J, et al (2006) Group M-based HIV-1 Gag peptides are frequently targeted by T cells in chronically infected US and Zambian patients. Aids 20: 353–360. 16439868 10.1097/01.aids.0000206501.16783.67

[53] Bijker MS, van den Eeden SJF, Franken KL, Melief CJM, Offringa R, et al (2007) CD8(+) CTL priming by exact peptide epitopes in incomplete Freund’s adjuvant induces a vanishing CTL response, whereas long peptides induce sustained CTL reactivity. Journal of Immunology 179: 5033–5040. 10.4049/jimmunol.179.8.5033 17911588

[54] Zhang GL, Srinivasan KN, Veeramani A, August JT, Brusic V (2005) PREDBALB/c: a system for the prediction of peptide binding to H2(d) molecules, a haplotype of the BALB/c mouse. Nucleic Acids Research 33: W180–W183. 15980450 10.1093/nar/gki479 PMC1160239

[55] BenMohamed L, Bertrand G, McNamara CD, Gras-Masse H, Hammer J, et al (2003) Identification of novel immunodominant CD4(+) Th1-type T-cell peptide epitopes from herpes simplex virus glycoprotein D that confer protective immunity. Journal of Virology 77: 9463–9473. 12915561 10.1128/JVI.77.17.9463-9473.2003 PMC187395

[56] Liao H-X, Sutherland LL, Xia S-M, Brock ME, Scearce RM, et al (2006) A group M consensus envelope glycoprotein induces antibodies that neutralize subsets of subtype B and CHIV-1 primary viruses. Virology 353: 268–282. 17039602 10.1016/j.virol.2006.04.043 PMC1762135

[57] Suhrbier A (2002) Polytope vaccines for the codelivery of multiple CD8T-cell epitopes. Expert Review of Vaccines 1: 207–213. 12901559 10.1586/14760584.1.2.207

[58] Ishioka GY, Fikes J, Hermanson G, Livingston B, Crimi C, et al (1999) Utilization of MHC class I transgenic mice for development of minigene DNA vaccines encoding multiple HLA-restricted CTL epitopes. Journal of Immunology 162: 3915–3925. 10201910

[59] Fuller DH, Shipley T, Allen TM, Fuller JT, Wu MS, et al (2007) Immunogenicity of hybrid DNA vaccines expressing hepatitis B core particles carrying human and simian immunodeficiency virus epitopes in mice and rhesus macaques. Virology 364: 245–255. 17428516 10.1016/j.virol.2007.02.024 PMC6286304

[60] Ngumbela KC, Day CL, Mncube Z, Nair K, Ramduth D, et al (2008) Targeting of a CD8 T cell Env epitope presented by HLA-B*5802 is associated with markers of HIV disease progression and lack of selection pressure. Aids Research and Human Retroviruses 24: 72–82. 18275350 10.1089/aid.2007.0124

[61] Kiepiela P, Ngumbela K, Thobakgale C, Ramduth D, Honeyborne I, et al (2007) CD8(+) T-cell responses to different HIV proteins have discordant associations with viral load. Nature Medicine 13: 46–53. 10.1038/nm1520 17173051

[62] Haynes BF, Gilbert PB, McElrath MJ, Zolla-Pazner S, Tomaras GD, et al.. (2012) Immune-Correlates Analysis of an HIV-1 Vaccine Efficacy Trial. New England Journal of Medicine 366. 10.1056/NEJMoa1113425 PMC3371689 22475592

[63] Duerr A, Huang Y, Buchbinder S, Coombs RW, Sanchez J, et al.. (2012) Extended Follow-up Confirms Early Vaccine-Enhanced Risk of HIV Acquisition and Demonstrates Waning Effect Over Time Among Participants in a Randomized Trial of Recombinant Adenovirus HIV Vaccine (Step Study). Journal of Infectious Diseases 206. 10.1093/infdis/jis342 PMC3490694 22561365

[64] Barouch DH, O’Brien KL, Simmons NL, King SL, Abbink P, et al (2010) Mosaic HIV-1 vaccines expand the breadth and depth of cellular immune responses in rhesus monkeys. Nature Medicine 16: 319–U116. 10.1038/nm.2089 PMC2834868 20173752

[65] Letourneau S, Im EJ, Mashishi T, Brereton C, Bridgeman A, et al.. (2007) Design and Pre-Clinical Evaluation of a Universal HIV-1 Vaccine. Plos One 2. 10.1371/journal.pone.0000984 PMC1991584 17912361

[66] Chevalier MF, Juelg B, Pyo A, Flanders M, Ranasinghe S, et al (2011) HIV-1-Specific Interleukin-21(+) CD4(+) T Cell Responses Contribute to Durable Viral Control through the Modulation of HIV-Specific CD8(+) T Cell Function. Journal of Virology 85: 733–741. 21047960 10.1128/JVI.02030-10 PMC3020027

[67] Nakanishi Y, Lu B, Gerard C, Iwasaki A (2009) CD8(+) T lymphocyte mobilization to virus-infected tissue requires CD4(+) T-cell help. Nature 462: 510–U205. 19898495 10.1038/nature08511 PMC2789415

[68] Shedlock DJ, Shen H (2003) Requirement for CD4 T cell help in generating functional CD8 T cell memory. Science 300: 337–339. 12690201 10.1126/science.1082305

[69] Almeida JR, Price DA, Papagno L, Arkoub ZA, Sauce D, et al (2007) Superior control of HIV-1 replication by CD8(+) T cells is reflected by their avidity, polyfunctionality, and clonal turnover. Journal of Experimental Medicine 204: 2473–2485. 17893201 10.1084/jem.20070784 PMC2118466

[70] Boaz MJ, Waters A, Murad S, Easterbrook PJ, Vyakarnam A (2002) Presence of HIV-1 gag-specific IFN-gamma+IL-2(+) and CD28(+)IL-2(+) CD4 T cell responses is associated with nonprogression in HIV-1 infection. Journal of Immunology 169: 6376–6385. 10.4049/jimmunol.169.11.6376 12444145

[71] Emu B, Sinclair E, Favre D, Moretto WJ, Hsue P, et al (2005) Phenotypic, functional, and kinetic parameters associated with a apparent T-cell control of human immunodeficiency virus replication in individuals with and without antiretroviral treatment. Journal of Virology 79: 14169–14178. 16254352 10.1128/JVI.79.22.14169-14178.2005 PMC1280210

[72] Sui YJ, Zhu Q, Gagnon S, Dzutsev A, Terabe M, et al (2010) Innate and adaptive immune correlates of vaccine and adjuvant-induced control of mucosal transmission of SIV in macaques. Proceedings of the National Academy of Sciences of the United States of America 107: 9843–9848. 20457926 10.1073/pnas.0911932107 PMC2906837

